# Editorial of Special Issue “Differential Diagnosis for Dry Eye”

**DOI:** 10.3390/diagnostics11050910

**Published:** 2021-05-20

**Authors:** Georgi As. Georgiev, Norihiko Yokoi

**Affiliations:** 1 Biointerfaces and Biomaterials Laboratory, Department of Optics and Spectroscopy, School of Optometry, Faculty of Physics, St. Kliment Ohridski University of Sofia 5 James Bourchier Blvd., 1164 Sofia, Bulgaria; 2 Institute for Bioengineering and Biosciences, Instituto Superior Técnico, Universidade de Lisboa, 1649-004 Lisbon, Portugal; 3 Department of Ophthalmology, Kyoto Prefectural University of Medicine, Kyoto 602-8566, Japan; nyokoi@koto.kpu-m.ac.jp

This editorial aims to summarize the scientific papers that contributed to the Special Issue “Differential Diagnosis for Dry Eye”.

The ocular surface communicates with the surrounding environment via the tear film (TF), a delicate wetting film composed of tear film lipid layer (TFLL) at the air/tear surface and underlying aqueous tear (AT) situated over the glycocalyx of the corneal epithelium. When some of the TF layers are quantitatively or qualitatively impaired, this results in the development of dry eye disease (DED) characterized by tear film (TF) instability and ocular discomfort and/or visual impairment. DED is the most prevalent public health ophthalmic disease affecting the quality of life of 10–30% of the human population worldwide, thus exerting a gross socioeconomic impact on modern society. A major reason for DED prevalence is that it can be caused by a plethora of factors characteristic of modern life [1]: relatively low air humidity and the non-optimal air temperature of air-conditioned offices, substandard illumination, contact lens wear, staring at displays etc. Furthermore, the workspace of many industries, like garment factories and other manufacturing industries [2], poses inherent occupational risk factors (dust particles, aerosols, processing chemicals, etc.) facilitating TF instability and the development of dry eye. Another factor that contributes to the increasing prevalence of DED in developed countries is the general aging of the population. In particular, it was found [3] that age is a determining factor of dry eye-related signs and symptoms “with younger subjects showing severe non-visual symptoms with apparently normal tear function and severe keratopathy, and older subjects showing fewer symptoms and less severe keratopathy despite worse tear function”.

DED is a multifactorial disease that may involve insufficiency in any of the TF layers, lipid and/or mucoaqueous, or in the ocular surface epithelium. While the impact of TFLL-related abnormalities has received plenty of attention in recent years, there is a growing body of evidence that quantitative or qualitative deficiencies of the secretory and membrane associated mucins (i.e., a topic far less studied up to now) may significantly impair TF stability. The quantitative determination of MUC5AC, the main secretory mucin in AT, has posed significant challenges due to inter- and intra-individual variability and small sample volumes. Hence the approach proposed by Woodward et al. [4] represents significant progress in this direction as it allows for short-term reproducibility of MUC5AC measurement in human tear fluid in spite of the high inter-individual variability. Regarding membrane-associated mucins, it is assumed that corneal fluorescein staining may report on their deficiency, as well as the overall structure of the corneal epithelium surface. Here, it is reported that patchy pattern corneal staining is correlated with dry eye disease and may indicate Sjögren’s syndrome or ophthalmological characteristics compatible with Sjögren’s syndrome [5].

TF instability can be caused by completely distinct underlying reasons and mechanisms. Also DED can be associated with a plethora of other confounding pathologies—like granulomatosis with polyangiitis (GPA) [6], systemic dehydration [7], conjunctivochalasis [8], etc.—and can serve as a potential reporter for these conditions in clinical practice. Thus in order for the best personalized therapy to be delivered to the patient, a differential diagnosis able to identify the root cause(s) of the disease is necessary. One technique that has gained popularity in the last decade is the measurement of tear osmolarity, as it requires small (submicroliter) sample volume and allows for a rapid measurement. A very interesting potential application discussed in great detail by Bron and Willshire [7] is to use tear osmolarity as a marker for systemic dehydration, a condition that is common in the elderly and results in high morbidity and significant mortality. Thus, timely DED diagnostics can serve as a live-saving diagnostics marker in the elderly population. However, the osmolarity measurements are not lacking challenges [9] as (i) they suffer from significant inter- and intra-individual variability and (ii) require expensive equipment as the values obtained with hand-held device do not correlate with any subjective or objective parameters of DED. Another approach developed by Itokawa et al. [10] probes the correlation between temperature and blood flow in the ocular anterior segment, and their effects on corneal temperature which is in turn related to the overall stability of the tear film. It is an interesting attempt that requires further studies in order to evaluate its clinical value.

A major diagnostics approach developed [11] in recent years is the analysis of the characteristics of fluorescein break-up patterns (FBUPs) which may allow for differentiation of different dry eye subtypes based on their underlying pathological mechanism, which in turn allows for TF-oriented therapy optimized for the treatment of the individual patients. Another important aspect is that, apart from differences in clinical features, the different FBUP types of DED also differ in terms of subjective discomfort of the patient and it is important to know how these aspects correlate each with other. Therefore, analysis of eye pain between dry eye (DE) subtypes using questionnaires and the PainVision^®^ (Osachi) apparatus was performed [12] and revealed that greater severity of eye pain was found in aqueous-deficient DE and decreased wettability DE than in increased evaporation DE.

These two studies [11,12] represent a very important clinically applicable development of the FBUPs diagnostic test for DED subgroups and are of immediate interest both for the clinician and for the basic scientist in the field of dry eye and tear film studies.

## Dedication

Georgi As. Georgiev dedicates this special issue to the memory of his beloved mother Emi Petrova Markova (1946–2021). Coming from a humble background Emi was the greatest mother I can dream of. She dedicated her entire life and unconditional love and support to make me into a successful person. She always believed in me and gave me the inspiration and faith that were essential for everything meaningful I have achieved in life, both in science and elsewhere.
